# Remedies and Their Uses

**Published:** 1906-10-27

**Authors:** 


### Remedies and their Uses.

### Quinine Compounds.

The use of quinine in medicine is undoubtedly somewhat restricted by its unpleasant taste. Especially in the case of young children, the bitter taste almost contra-indicates its employment; though, on the other hand, in small quantities combined in an acid mixture it forms a favourite "pick-me-up" and bitter tonic. In whooping cough quinine has been found useful by many physicians, a convenient formula being:—

| | |
|---|---|
| ℞ Quininæ hydrochloridi ... ... ... | gr. ½ |
| Tr. belladonnæ ... ... ... ... | ♏iij. |
| Syrupi tolutani ... ... ... ... | ♏v. |
| Aq. carui ... ... ... ... ... | ad ℥j |

for a child of a year old. The earliest attempts at producing a tasteless quinine consisted in substituting cinchonine, which, though not so bitter, is also much less reliable in its action. Various salts of quinine were then tried, the favourite for some time being the tannate, which, owing to its insolubility, is practically tasteless. Its action is, however, slow and not very sure, as its decomposition does not begin till the drug has entered the intestine. Recently the quinine molecule has been somewhat differently treated; the object has been to form an ester which shall be both tasteless and free from unpleasant by-effects.

These esters are tasteless merely owing to their insolubility; their salts, if soluble, are bitter, just as quinine salts; whereas the insoluble salts of the esters are tasteless, as for instance euquinine tannate.

*Aristoquin.*

This is the carboxylic acid ester of diquinine. It is practically insoluble and therefore tasteless. Besides its application in malaria and whooping cough, this substance has been found of value in allaying the cough of asthma and bronchitis, when given in doses of gr. vj. In nervous diseases, such as neuralgia (facial, intercostal, etc.) doses of about gr. xv. are necessary. Generally speaking, the dose should be higher than would be the case with quinine hydrochloride. A remarkable property of aristoquin is its solubility in dilute acids. It is immediately dissolved in the stomach, but is not reprecipitated in the intestine; the excretion in urine during the first twenty-four hours is considerable, though not so high as that of the ordinary hydrochloride. It has a bactericidal power which is higher than quinine, but its toxic effect on man, if judged by the influence on the blood-pressure, is not so high. It is recommended in cases where the secretion of HCl in the stomach is reduced, that after the aristoquin has been completely swallowed a drink of dilute HCl should be taken flavoured with lemon syrup. Aristoquin is manufactured by Bayer, of Elberfeld.

*Euquinine.*

This is an ester of quinine and carboxylic acid. It is practically tasteless. It is efficacious in malaria, but it is interesting to note that one observer (Budberg) finds it necessary to give large doses (at least gr. xv.) in good time before the attack is expected (about six hours). This is no doubt due to the insolubility. For whooping cough Kramer has formulated the following dosage: first year, gr. j.; second year, gr. iij.; third year, gr. vi.; fourth year, gr. viij.; fifth year, gr. ix.; sixth and seventh years, gr. xj.; eighth year, gr. xij.; ninth and tenth years, gr. xv. These doses may be given up to four times daily. Enquinine is said to stimulate the appetite, but without setting up gastro-intestinal irritation. It is prepared by E. Merck, of Darmstadt.

*Saloquinine.*

Salicylic acid has also been combined with quinine to form an ester. This body is tasteless and insoluble in water, but soluble in dilute hydrochloric acid. Animal experiments show that the salicylic acid does not reappear as such in the urine. Saloquinine, besides being tasteless, is said to produce more of the unpleasant by-effects of quinine itself. Its action, however, appears to be less marked therapeutically, and it is chiefly recommended in neuralgia and whooping cough. Adults can take 15 to 30 grains once or oftener daily; 1 to 4 grains may be given, according to age, to children with whooping cough every four or every six hours. As with the previous salt, dilute HCl may be subsequently given to facilitate the solution of the drug in the stomach. A salicylate of saloquinine has also been prepared and is said to be a useful drug in acute rheumatism and other forms of arthritis and neuritis. It combines the effects of salicylic acid with those of quinine. The dose is 45 grains daily for three days; then for a day it is omitted, and then resumed for four days, 60 grains being given. After this a pause is made every fifth day. It has the advantage over its two component substances of being absolutely tasteless; it is with difficulty dissolved in water. Both saloquinine and its salicylate are manufactured by Bayer, of Elberfeld.

